# Sleeping Beauty? A Prospective Study on the Prevalence of Sleep Problems and Their Potential Determinants in Professional Dancers

**DOI:** 10.1186/s40798-024-00798-2

**Published:** 2024-12-20

**Authors:** Astrid Junge, Rogier M. van Rijn, Janine H. Stubbe, Anja Hauschild

**Affiliations:** 1 Center for Health in Performing Arts, MSH Medical School Hamburg, Am Kaiserkai 1, 20457 Hamburg, Germany; 2 Institute of Interdisciplinary Exercise Science and Sports Medicine, MSH Medical School Hamburg, Hamburg, Germany; 3 Codarts Rotterdam, University of the Arts, Rotterdam, The Netherlands; 4 Performing Artist and Athlete Research Lab (PEARL), Rotterdam, The Netherlands; 5 Center for Rehabilitation and Sports Medicine, BG Klinikum Hamburg, Hamburg, Germany

**Keywords:** Sleep disturbance, Mental health, Workload, Ballet, Performing artists

## Abstract

**Background:**

Sleep is important for health and performance but has rarely been studied in professional dancers. The aim was to analyse the prevalence of sleep problems in professional dancers and their potential determinants at the beginning of and during the season.

**Methods:**

Professional dancers of six German companies answered a comprehensive baseline questionnaire on physical and mental health, including the Sleep Difficulty Score of the Athletic Sleep Screening questionnaire (ASSQ-SDS) in the beginning of the season and weekly health reports during the season. Numerical rating scales were used for severity of poor sleep, musculoskeletal pain, being stressed/overloaded, all health problems, impaired ability to dance, and workload in the previous seven days.

**Results:**

Of the 147 dancers who answered the baseline questionnaire, 104 (70.7%) completed in total 3186 weekly health reports (response rate: 71.2%). In the beginning of the season 53% of the dancers reported sleep problems of mild (34.0%), moderate (13.6%) or severe extent (5.4%), without differences between sexes, age groups, ranks of the dancers, company sizes or dance styles. The average weekly prevalence of “poor sleep” during the season was 68.8%. Multivariate regression analyses showed that symptoms of depression were significant determinants of sleep problems and the rating of “poor sleep” at baseline; while musculoskeletal pain, being stresses/overloaded, all health problems, impaired ability to dance, and physical and mental workload were significant determinants of “poor sleep” during the season. Variables of the baseline questionnaire were not significantly related to the individual mean rating of “poor sleep” during the season, except of the quality and duration of sleep.

**Conclusion:**

Sleep problems are frequent in professional dancers and related to their physical and mental health and workload. An assessment of sleep should be part of routine health screenings and interventions to improve sleep should be implemented, especially for dancers with pre-existing sleep problems and for periods of high workload.

## Key Points

• In the beginning of the season 53% of the dancers reported sleep problems without differences between sexes, age groups, ranks of the dancers, and companies of different size or dance style.

• The severity of sleep problems in the beginning of the season was on average higher in dancers with more severe symptoms of depression and of generalised anxiety disorder, current need for psychotherapeutic support as well as with more severe ratings of musculoskeletal pain, being stressed/overloaded, all health problems and impaired dance ability.

• The dancers reported moderate or severe sleep problems in 27.2% of the weeks of the season. Sleep quality during the season was related to the physical and mental health and workload in the same week.

## Background

Sleep is important for physical and mental health [1–4] and athletic performance [5], but sleep problems have rarely been investigated in dancers and these studies varied substantially in their aims, study design, methods, and results.

The prevalence of sleep problems was 63.5% [6] and 50% [7] in two studies on professional dancers and ranged between 80.1% [8] and 27.3% [9] in four studies on collegiate dancers or dance students. In a study [6] on 96 professional dancers 63.5% had sleep disturbances based on the Athlete Sleep Screening Questionnaire (ASSQ), while in another study [7] 50% of 24 professional ballet dancer showed signs of sleep disturbance according to the Pittsburgh Sleep Quality Index (PSQI). Using also the PSQI, the prevalence of poor sleep quality was 81.4% in a study [8] on 114 dance students and 59.5% in another study [10] on 116 dance students. A study [11] on 72 collegiate dancers reported that 60% had poor sleep behaviour based on the Athlete Sleep Behavior Questionnaire (ASBQ), while in another study [9] on 198 collegiate dancers 27.3% reported trouble sleeping. The differences in prevalence between these studies may be partly due to differences in assessment methods and how sleep problems were categorized. For example, in the study by Junge & Hauschild [6] 63.5% of the dancers had sleep problems to some degree but only 22.9% had a clinically meaningful sleep disturbance.

Sleep disturbance can be a symptom of mental health problems, but it can also lead to ill mental health, especially anxiety and depression [3]. It has been shown that the extent of sleep disturbance of professional dancers correlated with the severity of their depression and generalised anxiety symptoms [6] and that male and female dancers with sleep problems had higher depression scores, and male dancers with sleep problems had also higher generalised anxiety scores [12].

In addition, Arbinaga et al. [8] found in a study on 114 dance students a strong link between poor sleep quality and psychological inflexibility - the difficulty in managing unpleasant emotions. Higher levels of psychological inflexibility were positively correlated with conditions like anxiety, depression, and sleep disorders, and negatively associated with quality of life, perceived health, and positive emotional experiences. Students with high psychological inflexibility also tend toward an evening chronotype, making a consistent, healthy sleep routine less likely, while those with greater psychological flexibility reported more morning-oriented patterns, which align with healthier lifestyle habits.

Further, a cross-sectional study in 54 professional ballet dancers showed that absence due to injury was correlated with sleep disturbances measured with the PSQI [13], while a prospective study on 72 collegiate dancers over a 7-month period did not reveal a consistent relation between injury and sleep assessed with the ASBQ [11]. The latter study [11] found that sleep and dance exposure hours were only correlated during the month when dancers had two performance weeks. Fietze et al. [7] observed in 24 professional ballet dancers that total sleep time and sleep efficiency assessed by wrist actigraphy decreased during preparation for a premier.

In summary, the information on sleep problems of professional dancers is limited but there are indications that sleep problems are frequent in dancers and may be related to their physical and mental health and workload during the season. Such findings could be of great importance for the protection of physical and mental health of professional dancers. Therefore, the primary aim of the present study was to assess the prevalence of sleep problems in the beginning of and during the season. The secondary aim was to analyse the association of sleep problems with physical and mental health in the beginning of and during the season as well as with workload during the season. Our hypothesis was that the extend of sleep problems correlates with health problems and workload.

## Methods

All dancers of six German opera houses or state theatres (*n* = 279) were asked to complete online (a) a comprehensive baseline questionnaire in the beginning of the season 2022/23 [12] and (b) weekly health reports using the Performing artist and Athlete Health Monitor (PAHM) [14, 15] during 43 weeks of the season (September 2022 to June 2023). All dancers had a professional dance education and were full time employed at their company. Two companies had less than twenty dancers, one company between twenty and fifty, and three more than fifty dancers. Four companies danced primarily ballet, one company contemporary and one revue. All dancers were informed about the content and aims of the study prior to its start, and those participating gave their written informed consent. The study was reviewed and approved by the ethical committee of the MSH Medical School Hamburg, Germany (MSH 2021/137). The study was conducted in accordance with the Declaration of Helsinki.

The **baseline questionnaire** included questions on age, sex, rank in the company, size and dance style of the company, previous or current need of psychotherapeutic support, the depression module of the Patient Health Questionnaire (PHQ-9) [16], the Generalized Anxiety Disorder questionnaire (GAD-7) [17], and the five questions of the Athlete Sleep Screening Questionnaire (ASSQ-SDS) [18, 19] that sum up to the Sleep Difficulty Score (ASSQ-SDS).

The Athlete Sleep Screening Questionnaire (ASSQ) [18] was developed to identify athletes with a clinically significant sleep disturbance. Answers to five questions of the ASSQ (sleep duration, quality, problems falling asleep and staying asleep, sleep medication) are added up to the ASSQ-SDS, which was validated by Bender et al. [19]. Based on the ASSQ-SDS sleep problems are categorized as none (0–4), mild (5–7), moderate (8–10) and severe (11–17). Moderate and severe sleep problems were classified as clinically meaningful [19]. The ASSQ-SDS demonstrated good agreement with the rating of a sleep medicine physician based on a standardized clinical sleep interview (Cohen’s kappa = 0.84) [19]. The diagnostic sensitivity of the ASSQ-SDS was 81%, the specificity 93%, the positive predictive value 92%, and the negative predictive value 90% for clinical meaningful sleep problems [19].

Both **the baseline questionnaire and the weekly health reports** included numerical rating scales (NRS) on the severity of poor sleep, musculoskeletal pain, being stressed/overloaded, all health problems ranging from “not at all” (0) to “worst imaginable” (10) and impairment of the ability to dance at full potential due to health problems ranging from “dance at full potential” (0) to “unable to dance” (10) in the previous seven days. The responses were categorized as no (NRS = 0), mild (NRS = 1–3) moderate (NRS = 4–6) or severe (NRS = 7–10). The **weekly health reports** included additional NRSs on physical and mental workload ranging from “much too low” (-5) over “ideal” (0) to “much too high” (+ 5). Workload was classified as “lower than ideal” (NRS < 0), “ideal” (NRS = 0) or “higher than ideal” (NRS > 0).

The baseline questionnaire and the weekly health reports were pseudonymised (i.e. dancers used a personal code) to match the data. Only the individual dancer and the last author of the study knew the match of code and name and kept this information strictly confidential and in accordance with the German data protection laws.

### Statistical Analysis

All data were processed using Excel (version 16.74) and SPSS (version 27). Statistical methods applied were frequencies, descriptives, chi^2^-test, t-test, ANOVA, and multivariate linear regression analysis. Four regression analysis were calculated to predict (a) the ASSQ-SDS in the beginning of the season, (b) the severity rating of “poor sleep” score in the beginning of the season, and (c) the average severity rating of “poor sleep” during the season using variables of the baseline questionnaire, as well as (d) the severity rating of “poor sleep” during the season using ratings of physical and mental health and workload in the same week of the season. Significance was accepted at *p* < .05. The level of significance for the analysis of potential determinants was corrected for multiple testing according to Bonferroni.

## Results

### Study Population

The baseline questionnaire was answered by 147 dancers of whom 104 dancers (70.7%) filled in at least 25% of the weekly health reports during the season. At the start of the season the 81 female (55.1%) and 66 male (44.9%) dancers were on average 27.1 years old (sd = 5.3, median 26.0, range 18–42 years) without difference between sexes. Most dancers danced classical ballet (*n* = 107, 72.8%), 13 (8.8%) contemporary and 27 (18.4%) revue. More than half of the participating dancers (*n* = 89, 60.5%) were employed in companies with more than fifty dancers, thirty (20.4%) in a medium size company and 28 (19.0%) in companies with less than twenty dancers.

### Sleep Problems in the Beginning of the Season

**Fig. 1:**
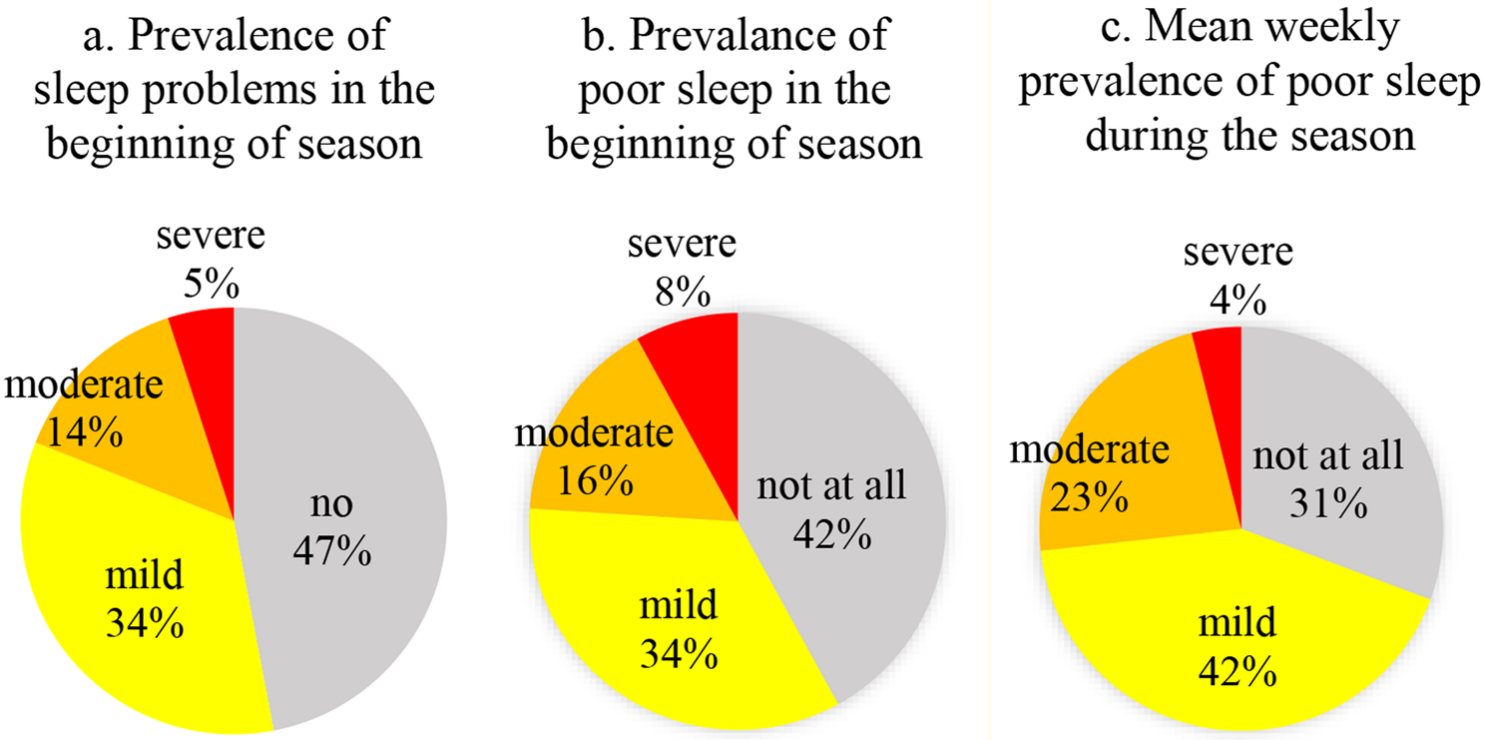
(**a**): Prevalence of differed severity of sleep problems (ASSQ-SDS) in the beginning of the season. ASSQ-SDS: Sleep Difficulty Score of the Athletics Sleep Screening Questionnaire, for definition of categories see methods section. Figure 1 (**b**) and (**c**): Prevalence of differed severity of poor sleep in the last 7 days on numerical rating scales (NRS) ranging from “not at all” (0) to “worst imaginable” (10) in the beginning of the season and during the season (not at all: NRS = 0; mild: NRS = 1 to 3; moderate: NRS = 4 to 6; severe: NRS = 7 to 10)

All dancers answered the ASSQ-SDS and 144 (98.0%) rated the severity of “poor sleep” in the beginning of the season. Most dancers reported to sleep either “seven to eight” (*n* = 69, 46.9%) or “six to seven” (*n* = 49, 33.3%) hours per night. Few dancers slept “five to six” (*n* = 12, 8.2%), “eight to nine” (*n* = 14, 9.5%) or “more than nine” (*n* = 3, 2.0%) hours per night. Almost all dancers (*n* = 133, 90.5%) fell asleep within 30 min or less, and had no (*n* = 68, 58.5%) or just once or twice per week (*n* = 37, 25.2%) trouble staying asleep. Most dancers were “very satisfied” (*n* = 32; 21.8%) or “somewhat satisfied” (*n* = 54, 36.7%) with their sleep quality, 25 (17%) “somewhat dissatisfied”, eight “very dissatisfied” (5.4%), and 25 (17%) answered “neither nor”. The majority (*n* = 131, 89.1%) never took sleep medication, nine (6.1%) once or twice per week, and seven (4.8%) more often. Based on the ASSQ-SDS, less than half of the dancers (*n* = 69, 46.9%) were classified as having no sleep problems, 34.0% mild (*n* = 50), 13.6% moderate (*n* = 20) or 5.4% severe problems (*n* = 8). The rating of “poor sleep” in the last seven days and the ASSQ-SDS correlated moderate to strongly (*r* = .64, *p* < .01) and led to similar prevalences (Fig. 1).

The prevalence of sleep problems was similar in different sexes, age groups, ranks of the dancers, and in companies of different sizes or dance styles. The prevalence of moderate to severe sleep problems ranged from 8.3% to 25.9% between the six companies but the difference did not reach statistical significance.

Comparisons of the mean ASSQ-SDS and the mean severity rating of “poor sleep” of dancers with different characteristics are presented in Table 1a and b. The ASSQ-SDS differed significantly between dancers with different severity of depression symptoms, symptoms of generalised anxiety disorder, and being stressed/overloaded. The average severity rating of “poor sleep” differed in the same variables and also in the severity of musculoskeletal pain and all health problems. All differences were in the expected direction, i.e. more sleep problems and poorer sleep was associated with the higher severity of other symptoms.

**Table 1:** Comparison of professional dancers with different characteristics in relation to the Sleep Difficulty score of the Athlete Sleep Screening Questionnaire (ASSQ-SDS) and to the rating of “poor sleep” in the past 7 days in the beginning of the season. Results significant after Bonferroni correction (*p* < .006) are highlighted in bold

	Sleep difficulty score (ASSQ-SDS)	Rating of poor sleep	
**Variable** Category	Comparison Mean (sd)	Comparison Mean (sd)	
**Symptoms of depression**	**F = 10.1, p < 0.001**	**F = 18.55, p < 0.001**	
No or (PHQ-9 < 5) (*n* = 106) Mild (PHQ-9 5–9) (*n* = 25)	4.44 (2.31) 6.48 (2.31)	1.16 (1.63) 3.56 (2.66)	
Moderate (PHQ-9 10–14) (*n* = 10) Severe (PHQ-9 > = 15) (*n* = 3)	7.80 (3.97) 8.33 (6.43)	1.16 (1.63) 3.56 (2.66)	
**Symptoms of generalised anxiety disorder**	**F = 5.08, p = 0.002**	**F = 7.04, p < 0.001**	
No (GAD-7 < 5) (*n* = 98) Mild (GAD-7 5–9) (*n* = 29)	4.55 (2.39) 6.00 (2.95)	1.31 (1.81) 2.90 (2.76)	
Moderate (GAD-7 10–14) (*n* = 10) Severe (GAD-7 > = 15) (*n* = 7)	7.30 (3.06) 6.14 (4.41)	3.20 (3.46) 4.00 (3.79)	
**Need for psychotherapeutic support**	F = 3.22, p = 0.043	F = 4.16, p = 0.018	
No (*n* = 55)	4.93 (2.60)	1.35 (2.06)	
Previously (*n* = 51)	4.65 (2.32)	1.92 (1.89)	
Currently (*n* = 39)	6.08 (3.44)	2.82 (3.32)	
**Severity of musculoskeletal pain** ^**#**^	F = 4.45, p = 0.011	**F = 9.81, p < 0.001**	
No or mild (NRS 0–3) (*n* = 86)	4.73 (2.48)	1.40 (2.01)	
Moderate (NRS 5–6) (*n* = 41)	5.49 (2.59)	2.32 (2.39)	
Severe (NRS 7–10) (*n* = 17)	6.88 (4.15)	4.06 (3.49	
**Severity of being stressed/overloaded** ^**#**^	**F = 6.26, p = 0.002**	**F = 16.06, p < 0.001**	
No or mild (NRS 0–3) (*n* = 104)	4.74 (2.38)	1.31 (1.78)	
Moderate (NRS 5–6) (*n* = 23)	6.91 (3.63)	3.65 (2.96)	
Severe (NRS 7–10) (*n* = 17)	5.65 (3.26)	3.71 (3.46)	
**Severity of all health problems** ^**#**^	F = 2.51, p = 0.085	**F = 6.02, p = 0.003**	
No or mild (NRS 0–3) (*n* = 87)	4.82 (2.55)	1.44 (2.02)	
Moderate (NRS 5–6) (*n* = 38)	5.39 (3.05)	2.47 (2.61)	
Severe (NRS 7–10) (*n* = 17)	6.41 (3.32)	3.41 (3.48)	
**Severity of impaired dance ability** ^**#**^	F = 4.57, p = 0.012	F = 4.8, p = 0.010	
No or mild (NRS 0–3) (*n* = 112)	4.80 (2.61)	1.65 (2.18)	
Moderate (NRS 5–6) (*n* = 20)	6.00 (2.77)	2.65 (2.41)	
Severe (NRS 7–10) (*n* = 12)	7.00 (3.81)	3.67 (3.99)	

ASSQ-SDS: Sleep Difficulty Score of the Athlete Sleep Screening Questionnaire; PHQ-9: depression module of the Patient Health Questionnaire; GAD-7: Generalized Anxiety Disorder-7; NRS: numeric rating scale; #: in the last 7 days

Multivariate regression analysis (Table 2) confirmed that neither the size or dance style of the company, nor the age or rank of the dancers were related to the ASSQ-SDS or the severity rating of “poor sleep”. The models for the ASSQ-SDS (F = 2.83; *p* = .002) and “poor sleep” (F = 6.53; *p* < .001) were statistically significant and explained 14.0% resp. 33.0% (corrected R^2^) of the variance. Symptoms of depression were the only variable that contributed significantly to the variability of the ASSQ-SDS and to the variability of “poor sleep” (Table 2).

**Table 2:** Multivariate regression analysis on the Sleep Difficulty score of the athletic sleep screening questionnaire (ASSQ-SDS) and on the severity rating of “poor sleep” in the past seven days using variables of the questionnaire in the beginning of the season. Statistically significant results are highlighted in bold

	ASSQ-SDS in the beginning of the season							Rating of “poor sleep” in the beginning of the season								
Variable (answer choices)	Beta	SE	Lower CI	Upper CI		F	p	Beta	SE	Lower CI		Upper CI		F		p
Constant	1.707	2.161	-2.570	5.984	0.790		0.431	0.194	1.650	-3.073		3.460		0.117		0.907
Size of the company < 20 / 20–50 / >50 dancers	0.176	0.302	− 0.422	0.774	0.583		0.561	0.032	0.231	− 0.425		0.488		0.137		0.891
Type of dance Ballet / Contemporary / Revue	0.062	0.322	− 0.576	0.701	0.194		0.847	0.041	0.246	− 0.446		0.529		0.168		0.867
Age of dancer Years	0.031	0.048	− 0.064	0.126	0.642		0.522	− 0.009	0.037	− 0.081		0.064		− 0.232		0.817
Sex Female / male	0.548	0.478	− 0.398	1.494	1.146		0.254	0.723	0.365	0.001		1.446		1.981		0.050
Rank of dancer Principal dancer. soloist / Others	0.048	0.581	-1.101	1.197	0.083		0.934	− 0.628	0.443	-1.506		0.250		-1.416		0.159
Symptoms of depression (PHQ-9) Sum score	0.380	0.113	0.157	0.602	3.369		**0.001**	0.374	0.086	0.204		0.545		4.353		**< 0.001**
Symptoms of generalised anxiety disorder (GAD-7) Sum score	− 0.073	0.089	− 0.249	0.104	− 0.816		0.416	− 0.132	0.068	− 0.267		0.002		-1.948		0.054
Need for psychotherapeutic support No / Previously / Currently	0.038	0.318	− 0.591	0.667	0.121		0.904	0.254	0.243	− 0.226		0.735		1.048		0.297
Musculoskeletal pain^#^ Numerical rating scale 0–10	− 0.024	0.141	− 0.302	0.254	− 0.172		0.864	0.080	0.107	− 0.132		0.293		0.749		0.455
Being stressed/overloaded^#^ Numerical rating scale 0–10	− 0.035	0.121	− 0.275	0.205	− 0.289		0.773	0.223	0.093	0.040		0.407		2.412		0.017
All health problems^#^ Numerical rating scale 0–10	0.021	0.153	− 0.282	0.324	0.138		0.890	− 0.044	0.117	− 0.275		0.188		− 0.375		0.708
Impaired dance ability^#^ Numerical rating scale 0–10	0.132	0.131	− 0.127	0.390	1.008		0.315	0.008	0.100		− 0.189		0.206	0.084	0.933	

PHQ-9: Depression module of the Patient Health Questionnaire; GAD-7: Generalized Anxiety Disorder-7; ASSQ-SDS: Sleep Difficulty Score of the Athlete Sleep Screening Questionnaire^37^; #: in the last 7 days

### Sleep Problems During the Season

During the 43 weeks of the season, 104 dancers filled in a total of 3186 weekly health reports (response rate: 71.2%). The dancers reported no “poor sleep” in 31.2% of the weeks, mild problems in 42.5%, and moderate or severe sleep problems in 27.2% of the weeks (Fig. 1c). The average rating of “poor sleep” during the season (2.37, sd = 2.36) was slightly higher than in its beginning (1.97, sd = 2.5). The severity rating of “poor sleep” differed significantly between weeks with different severity of physical and mental health problems and with different extend of physical and mental workload (Table 3). All differences were in the expected direction, i.e. poorer sleep was associated with the higher severity of other symptoms and higher workload. Sleep problems were lowest in weeks with “ideal” physical and mental workload, similar in weeks with “lower than ideal” and with “ideal” workload, and most severe in weeks with “higher than ideal” workload (Table 3).

**Table 3:** Comparison of average weekly rating of “poor sleep” in groups with different severity ratings of physical and mental health problems and workload in the same week of the season. Results significant after Bonferroni correction (p *≤* .008) are highlighted in bold

	Average weekly rating of poor sleep during the season
**Variable** Category	Comparison Mean (sd)
**Severity of musculoskeletal pain** ^**#**^	**F = 258.1, p < 0.001**
No or mild (NRS 0–3) (*n* = 1982)	1.72 (1.89)
Moderate (NRS 5–6) (*n* = 1019)	3.29 (2.55)
Severe (NRS 7–10) (*n* = 169)	4.50 (3.02)
**Severity of stressed/overloaded** ^**#**^	**F = 414.4, p < 0.001**
No or mild (NRS 0–3) (*n* = 2268)	1.70 (1.97)
Moderate (NRS 5–6) (*n* = 731)	3.88 (2.30)
Severe (NRS 7–10) (*n* = 171)	4.79 (2.86)
**Severity of all health problems** ^**#**^	**F = 216.5, p < 0.001**
No or mild (NRS 0–3) (*n* = 2329)	1.88 (2.01)
Moderate (NRS 5–6) (*n* = 740)	3.62 (2.63)
Severe (NRS 7–10) (*n* = 98)	4.46 (3.32)
**Severity of impaired dance ability** ^**#**^	**F = 54.8, p < 0.001**
No or mild (NRS 0–3) (*n* = 1706)	1.98 (2.07)
Moderate (NRS 5–6) (*n* = 339)	3.33 (2.58)
Severe (NRS 7–10) (*n* = 137)	2.50 (2.56)
**Extend of physical workload** ^§^	**F = 61.02, p < 0.001**
Lower than ideal (NRS < 0) (*n* = 592)	2.03 (2.07)
Ideal (NRS 0) (*n* = 948)	1.89 (2.32)
Higher than ideal (NRS > 0) (*n* = 1613)	2.80 (3.41)
**Extend of mental workload** ^§^	**F = 99.8, p < 0.001**
Lower than ideal (NRS < 0) (*n* = 424)	1.78 (1.90)
Ideal (NRS 0) (*n* = 1104)	1.73 (2.24)
Higher than ideal (NRS > 0) (*n* = 1724)	2.90 (2.36)

NRS: numeric rating scale; #: NRS ranging from “not at all” (0) to “worst imaginable” (10); § NRS ranging from “much too low” (-5) over “ideal” (0) to much too high” (+ 5)

**Fig. 2:**
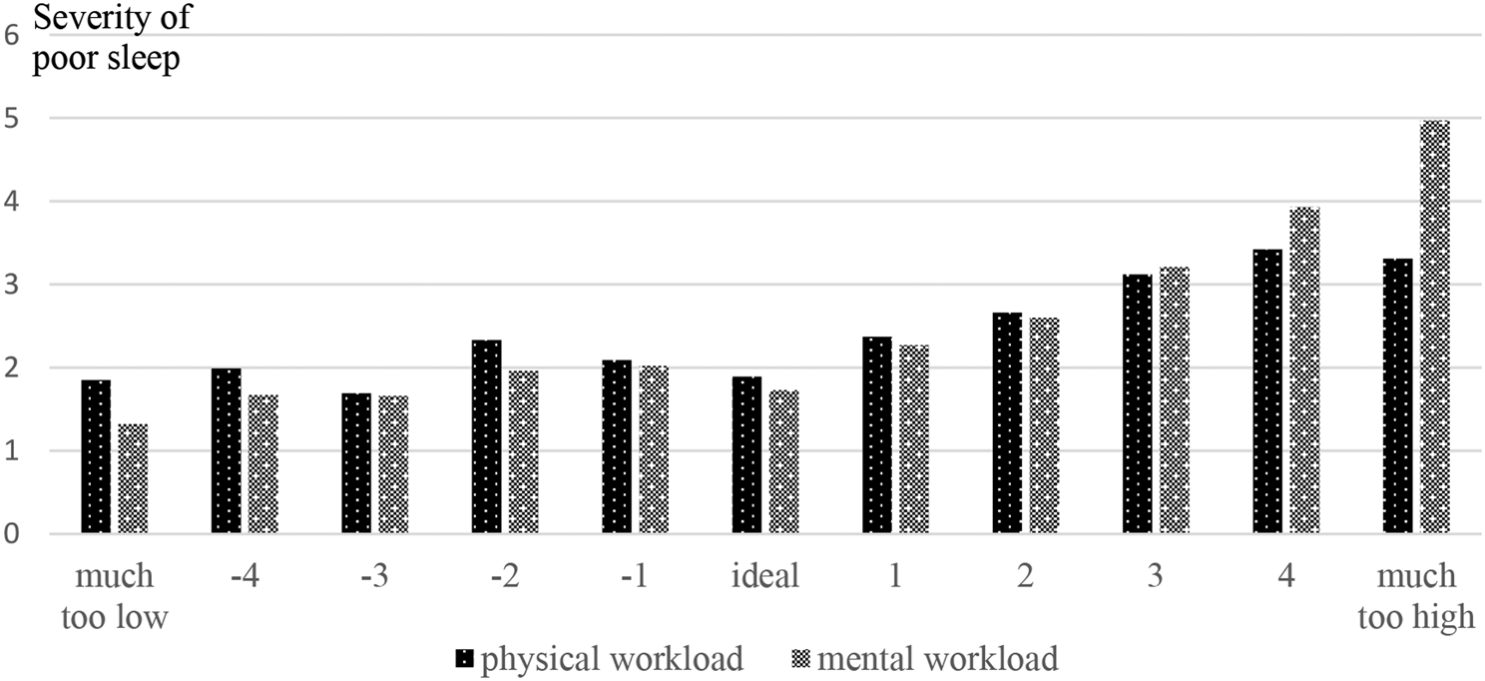
Average severity rating of poor sleep (numerical rating scales (NRS) ranging from “not at all” (0) to “worst imaginable” (10)) in weeks with differed rating of physical and mental workload in the last 7 days on NRS ranging from “much too low” (-5) over “ideal” (0) to “much too high” (+ 5)

A detailed analysis of the association of the ratings of “poor sleep” and workload (Fig. 2) supported this finding and demonstrated that the severity of sleep problems progressively increased when workload was rated “higher than ideal”. However, when dancers rated their physical workload as “much too high” sleep problems dropped slightly but were still second highest. The dancers´ sleep was poorest in weeks with “much too high” mental workload.

Multivariate regression analysis for severity rating of “poor sleep” during the season using the ratings of physical and mental health and workload in the same week (Table 4) was statistically significant (F = 200.7; *p* < .001) and explained 33.5% (corrected R^2^) of the variance. Severity ratings of musculoskeletal pain, being stressed/overloaded, all health problems, impaired ability to dance at full potential, physical and mental workload contributed significantly to the variability of “poor sleep”.

**Table 4:** Multivariate regression analysis of the weekly severity rating of “poor sleep” using ratings of physical and mental health problems and workload in the past 7 days of professional dancers during the season. Statistically significant results are highlighted in bold

	Rating of “poor sleep” during the season					
Variable (answer choices)	Beta	SE	Lower CI	Upper CI	F	p
Constant	0.658	0.062	0.537	0.779	10.672	< 0.001
Musculoskeletal pain^#^ Numerical rating scale	0.134	0.022	0.090	0.177	6.044	< 0.001
Being stressed/overloaded^#^ Numerical rating scale	0.368	0.020	0.330	0.407	18.780	< 0.001
All health problems^#^ Numerical rating scale	0.192	0.029	0.135	0.248	6.674	< 0.001
Impaired dance ability^#^ Numerical rating scale	− 0.075	0.022	− 0.119	− 0.032	-3.376	0.001
Physical workload§ Numerical rating scale	− 0.078	0.026	− 0.129	− 0.026	-2.966	0.003
Mental workload§ Numerical rating scale	0.061	0.029	0.006	0.117	2.154	0.031

#: Numerical Rating Scales (NRS) ranging from “not at all” (0) to “worst imaginable” (10); § NRS ranging from “much too low” (-5) over “ideal” (0) to much too high” (+ 5)

The multivariate regression model using the variables of the baseline questionnaire in the beginning of the season to predict the individual mean of “poor sleep” during the season was not significant and explained 2.6% of the variance with only duration and quality of sleep being significant variables.

## Discussion

To the best of our knowledge, this is the first prospective study that assessed the prevalence of sleep problems and their potential determinants in professional dancers in the beginning and during the season. In total, 147 professional dancers from six opera houses or state theatres answered the baseline questionnaire, of whom 104 dancers (70.7%) completed 3186 weekly health reports during the season. Almost 20% of the dancers had at least moderate sleep problems in the beginning of the season and slightly more during the season. Sleep problems in the beginning of the season were mainly explained by mental health symptoms, while poor sleep during the season was also influenced by physical health and workload. Variables of the baseline questionnaire did not explain the individual mean rating of “poor sleep” during the season, except of duration and quality of sleep in the beginning of the season.

### Prevalence of Sleep Problems

In agreement with previous studies [6, 7, 10, 11] more than half of the dancers in the present study reported sleep problems in the beginning of the season. During the season, sleep problems, especially of mild extent, were even more frequent. The prevalence of a clinically relevant sleep disturbance was 19% based on the ASSQ-SDS in the beginning of the season. The weekly prevalence of moderate to severe sleep problems based on the rating of “poor sleep” was 24% in the beginning of the season and 27% during the season. Similar prevalence rates have been reported from collegiate [9] and professional [6] dancers and from athletes practising various sports [19,20, 21]. However, it should be noted that the prevalence of a clinically meaningful sleep disturbance (ASSQ-SDS) ranged from 8.3 to 25.9% between the six companies.

### Factors Associated with Sleep Problems

The average severity of sleep problems was similar across sexes, age groups, ranks of the dancers, and companies of different sizes or dance styles. However, the ASSQ-SDS and the rating of “poor sleep” in the beginning of the season were higher in dancers with more symptoms of depression and of generalised anxiety disorder, need for psychotherapeutic support as well as with a higher severity of musculoskeletal pain, being stressed/overloaded, and impaired dance ability. The bi-directional relationship between mental health problems and sleep disturbances is well documented [3] and has been shown previously in professional dancers [6, 12]. Although obvious, less information was found on the association of pain and sleep [22] and no study analysed this relation in dancers previously. The two studies [11, 13] on the relation of injuries and sleep problems in dancers had inconsistent results.

In the present study, “poor sleep” during the season was associated with physical and mental health problems but also with physical and mental workload. Sleep quality was best in weeks with “ideal” or “lower than ideal” physical and mental workload and deteriorated gradually with increasing workload. The negative effect of a high density of performance and preparation for a premier has been demonstrated previously [7, 11]. In the present study, sleep quality was poorest in weeks with “much too high mental workload” but not in weeks with “much too high physical workload”, most probably because the dancers were then too exhausted.

When the characteristics of the dancers reported in the baseline questionnaire were used to predict the average individual sleep quality during the season, only the duration and quality of sleep were significant but not the ASSQ-SDS nor the mental or physical health in the beginning of the season. This indicates that pre-existing sleep characteristics may influence the sleep quality during the season. Therefore, it is recommended to include an assessment of sleep in routine health screenings and provide interventions to improve duration and quality of sleep to those affected by sleep problems.

### Strength and Limitations

The present study was part of a larger project which included a health and performance screening, a comprehensive baseline questionnaire, and weekly follow-ups throughout the season [12, 23]. All dancers of six companies were invited to participate in the study, but not all agreed for various reasons (e.g. too much workload, injury, confidentiality concerns). Therefore, it is likely that no selection bias has affected the results of the present study. Further, most previous studies included dancers from just one company or school and the present study showed that the prevalence of sleep problems varied substantially between companies. Although confidentiality was assured, it cannot be ruled out that some answers to physical and mental health questions were biased due to stigma, denial, or fear of potential consequences, but this limitation also applies to almost all studies on physical and mental health in dancers and other elite athletes. The analysis of sleep problems during the season was based just on the rating of “poor sleep” and not on an established questionnaire, but a longer weekly questionnaire would have substantially reduced compliance and we have shown that the rating highly correlated with the ASSQ-SDS.

## Conclusion

Sleep problems were frequent in professional dancers in the beginning of the season and during the season. Sleep problems in the beginning of the season were closely associated with physical and mental health. The severity of “poor sleep” during the season was also related to the physical and mental health and workload in the same week, and to the duration and quality of sleep in the beginning of the season. Therefore, an assessment of sleep should be part of routine health screenings and interventions to improve sleep duration and quality should be implemented, especially for dancers with pre-existing sleep problems and for periods of high workload.

## Data Availability

Due to confidentiality reasons, no data can be shared.

## Abbreviations

ANOVA: Analysis of variance
ASSQ: Athlete sleep screening questionnaire
ASSQ-SDS: Sleep difficulty score of the ASSQ
GAD-7: Generalized anxiety disorder-7
NRS: Numeric rating scale
PHQ-9: depression module of the patient health questionnaire
SD: Standard deviation
SPSS: Statistical package für social sciences
